# Identification of novel mitosis regulators through data mining with human centromere/kinetochore proteins as group queries

**DOI:** 10.1186/1471-2121-13-15

**Published:** 2012-06-19

**Authors:** Aaron R Tipton, Kexi Wang, Peter Oladimeji, Shermeen Sufi, Zhidong Gu, Song-Tao Liu

**Affiliations:** 1 Department of Biological Sciences, University of Toledo, Toledo, OH, 43606, USA; 2 Ruijin Hospital, Shanghai, 200025, China

**Keywords:** Centromere, Kinetochore, Centrosome, Data mining, Protein-protein interaction, Co-expression

## Abstract

**Background:**

Proteins functioning in the same biological pathway tend to be transcriptionally co-regulated or form protein-protein interactions (PPI). Multiple spatially and temporally regulated events are coordinated during mitosis to achieve faithful chromosome segregation. The molecular players participating in mitosis regulation are still being unravelled experimentally or using *in silico* methods.

**Results:**

An extensive literature review has led to a compilation of 196 human centromere/kinetochore proteins, all with experimental evidence supporting the subcellular localization. Sixty-four were designated as “core” centromere/kinetochore components based on peak expression and/or well-characterized functions during mitosis. By interrogating and integrating online resources, we have mined for genes/proteins that display transcriptional co-expression or PPI with the core centromere/kinetochore components. Top-ranked hubs in either co-expression or PPI network are not only enriched with known mitosis regulators, but also contain candidates whose mitotic functions are not yet established. Experimental validation found that KIAA1377 is a novel centrosomal protein that also associates with microtubules and midbody; while TRIP13 is a novel kinetochore protein and directly interacts with mitotic checkpoint silencing protein p31^comet^.

**Conclusions:**

Transcriptional co-expression and PPI network analyses with known human centromere/kinetochore proteins as a query group help identify novel potential mitosis regulators.

## Background

Mitosis is a complicated cellular process involving extensive structural reorganizations in many subcellular compartments and a sequence of highly orchestrated events. The temporal and spatial changes in mitotic cells are tightly regulated to ensure high fidelity of genomic transmission during cell division. Mitosis is initiated by accumulation of active kinase complexes formed between mitotic cyclins (cyclin A and B in human) and master mitosis regulator CDC2 (or CDK1) at the G2/M transition [1]. The expression of many other mitosis regulators also peaks during G2/M phase, some of which share the common control by transcription factors such as Fox M1 and the DREAM complex [2,3]. Chromosome condensation appears during prophase, concurring with reorganization of microtubule cytoskeleton into mitotic spindles and separation of duplicated centrosomes to opposite sides of the nucleus. The activity of cyclin A/CDC2 lasts until nuclear envelope breakdown when cyclin A is degraded [4]. The cyclin B/CDC2 complex, together with many other chromosome and microtubule associated proteins, promotes the formation of bipolar spindle and chromosome congression to the metaphase plate [1]. Cyclin B is then destructed after ubiquitylation by the anaphase promoting complex/cyclosome (APC/C) [1,5]. Loss of cyclin B/CDC2 activity ensures unidirectional progression of mitosis [6]. Sister chromatids separate and move to the opposite spindle poles after anaphase onset. Chromosomes then decondense, and the nuclear envelope reforms during telophase. When cytokinesis is completed, the abscission occurs at the midbody between two daughter cells, the spindle is disassembled and cells flatten out into interphase morphology again.

The centromeres are specialized loci on chromosomes that form primary constrictions during mitosis. There are currently 18 known human proteins constitutively associating with centromeres throughout the cell cycle [7]. The kinetochores are macromolecular protein complexes built upon centromeres to connect with spindle microtubules [8]. Kinetochores are dynamic structures that are assembled and disassembled at each sister chromatid during each and every mitosis [8]. Kinetochores also harbour activities contributing to chromosome movement throughout mitosis and the spindle assembly checkpoint (SAC) [5]. The SAC monitors kinetochore-microtubule attachment status to inhibit activation of the APC/C until the metaphase-to-anaphase transition. Under electron microscope centromeres and kinetochores are contiguous structurally, and both play important roles in regulating chromosome segregation. We will use the term “the centromere/kinetochore complex” in this paper to reflect the intertwined relationship between the two subcellular structures.

Although the knowledge of proteins localized at the centromere/kinetochore complex has increased exponentially in the past few years [7-11], we still have much to learn about the proteins that contribute to the spatial and temporal regulation of mitosis. As indicated in recent reports, without a “parts list” of critical molecular players, it is impossible to reach a comprehensive understanding about mitosis and its connections with tumorigenesis and cancer drug effects [9,10,12]. Recent genomics and proteomics research has revealed that proteins in the same functional modules are usually transcriptionally co-expressed and/or organized into clusters in PPI networks (e.g. [13-15]). Many publicly available bioinformatics resources have deposited data obtained from large scale co-expression profiling and PPI studies, and provided tools to retrieve and organize the data.

As an end user interested in taking advantage of the huge amount of genomics and proteomics data in the public databases, we first conducted exhaustive literature review and compiled a list of 196 human centromere/kinetochore proteins, and selected 64 among them as “core” components with well-characterized mitotic functions. By interrogating and integrating available online resources using the 64 core proteins as a query group, we have then identified potential novel mitosis regulators among top-ranking genes/proteins that co-express or interact with the 64 core centromere/kinetochore components. Experimental validation has identified one novel kinetochore protein TRIP13 and two novel centrosomal proteins KIAA1377 and DDX39.

## Results and discussion

### Compiling a comprehensive list of human centromere/kinetochore proteins

Several reviews have previously summarized known centromere/kinetochore proteins in human cells [7-11,16,17]. Due to rapid progress in the field and extensive use of aliases in literature, omissions in the published lists have become obvious; therefore we carried out an exhaustive literature search aiming to compile a comprehensive list of human centromere/kinetochore proteins. A protein is defined as centromere/ kinetochore-localized only when experimental evidence such as immunofluorescence or fluorescent protein fusions supported the claims (except several condensin and cohesin subunits, see below). As updated until April 30, 2012, a total of 196 human proteins correlating to specific genes have been localized at the centromere/kinetochore complex in published literature (Additional file 1: Table S1 and references therein). In addition, a phospho-specific epitope, recognized by monoclonal antibody 3 F3/2 [18], resides in kinetochore proteins. The epitope, generated at least partially by Plk1 kinase, is likely to be found at multiple centromere/kinetochore proteins [19,20]. Some proteins carrying 3 F3/2 epitopes have been determined (e.g. BUBR1) [21], but many remain to be characterized. Among all the centromere/kinetochore proteins, only two cohesin subunits encoded by *Rec8* and *STAG3* are meiosis specific. Not all condensin and cohesin subunits have been experimentally localized at the centromere/kinetochore complex, but both condensin and cohesin complexes are essential non-histone structural components along chromosomes, and play important roles in chromosome dynamics throughout the cell cycle, we therefore tentatively include all condensin and cohesin subunits as centromere/kinetochore proteins [22,23]. To facilitate future research on the centromere/kinetochore proteins, in Table S1 we included gene symbols, Entrez gene IDs and common aliases for each gene.

When compared to previous summaries, the compilation has significantly expanded the list of known human centromere/kinetochore proteins, from ~120 to 196. The list still did not include all the subunits of several well-characterized protein complexes such as the dynein-dynactin complex and the γ-tubulin ring complex, both shown to associate with the centromere/kinetochore [24]. Most likely the missing subunits are also targeted to the centromere/kinetochore as part of the protein complexes but as yet the localization has not been experimentally demonstrated. We will also report TRIP13 as a novel kinetochore protein below. A recent mass spectrometry based study estimated a total of ~200 kinetochore proteins [12]. Our survey indicates that the human centromere/kinetochore is indeed a complicated structure with constitutive and transient components easily exceeding 200 proteins.

We compared our list of human centromere/kinetochore proteins to annotations in Gene Ontology (GO) (http://www.geneontology.org/) [25]. The GO term search returned 49 categories containing “centromere” and 31 containing “kinetochore” in the GO titles or definitions. Not all these GO categories contain human proteins. A total of 247 human proteins have been annotated in GO to be involved in centromere- or kinetochore-related localization, functions or processes, of which 128 appeared in our list of centromere/kinetochore proteins (Additional file 2: Table S2). Among the remaining 119 GO annotated centromere/kinetochore proteins, some may participate in regulation of centromere/kinetochore functions but do not localize at the structure themselves (e.g. proteins encoded by *SUGT1* and *SENP6*) [26,27]. Some others such as *CENPBD1* were annotated by inference without experimental evidence. A few more genes (e.g. *BAZ1B*) have been localized in other species but not in human cells. Proteins listed in the later two categories are worthy of further exploration in order to completely catalogue human centromere/kinetochore components (Additional file 2: Table S2). However, *SS18L1* may be mistakenly annotated because the acronym of one of its alias, CREST, is identical to the commonly used autoimmune antibody to stain centromeres.

### Genes co-expressing with 64 core centromere/kinetochore components

Transcriptional co-expression profiling has been extensively used to uncover functional gene modules involved in common biological processes [15,28-33]. These modules can be detected through comparing the similarity of gene expression patterns using Pearson correlation coefficient or other metrics. Value-based and rank-based methods have been developed to construct the co-expression network. Advantages of the rank-based method have been discussed in recent publications [34,35].

In our initial efforts, we adopted a simple rank-based method to retrieve genes that co-express with a selection of 64 “core” centromere/kinetochore components (Table 1, also marked by asterisks in Additional file 1: Table S1). The 64 genes were chosen based on higher expression and/or better characterized functions in mitosis. The selection, when used collectively as a query group, is expected to reduce noises common in any association studies and enhance the specificity in retrieving mitosis-relevant genes. Upon querying the Human Gene Sorter with the 64 genes, we extracted and ranked a total of 3828 genes that appeared at least once in a combined list of “top 50” co-expressing genes (Additional file 3: Table S3, see Methods for details). Forty-six of the 64 queries were represented in the combined list, with a total of 422 occurrences (Table 1). Moreover, 111 of 196 known centromere/kinetochore proteins appeared 680 times in total. The representation of both groups was significantly higher than by chance (Z scores at 13.52 and 7.66 respectively, P < 0.001).

**Table 1:** The 64 core centromere-kinetochore components as queries for database searches

**Gene Symbol**	**Number of datasets in Gene Sorter**	**Occurrences in combined Gene Sorter "top 50" co-expression list**	**Notes**
APITD1	3	2	CENPS
AURKB	3		
BIRC5	3	1	
BUB1	3	20	
BUB1B	3	30	
BUB3	3		
C14orf106	2		Mis18BP1
C21orf45	2	1	Mis18A
C6orf173	1	4	CENPW
CASC5	2	3	KNL1
CCDC99	2	3	Spindly
CDC20	3	7	
CDCA8	2	19	Borealin
CENPA	3	14	
CENPB	3		
CENPC1	3		
CENPE	3	19	
CENPF	3	11	
CENPH	2	2	
CENPI	3		
CENPK	2	3	
CENPL	2		
CENPM	2	5	
CENPN	2		
CENPO	2	1	
CENPP	2		
CENPQ	2	2	
CENPT	2		
CENPV	2		PRR6
CLASP1	3	1	
CLASP2	3		
CLIP1	3		
DSN1	2		
ERCC6L	2	1	PICH
HJURP	2	9	
INCENP	2	1	
ITGB3BP	3	23	CENPR
KIF2C	3	23	
KNTC1	3	11	Rod
MAD1L1	3	1	
MAD2L1	2	11	
MAD2L1BP	3	2	p31comet
MIS12	2		
MLF1IP	2	20	CENPU
NDC80	3	12	
NEK2	3	13	
NSL1	2	1	
NUF2	2	7	
OIP5	3	20	MIS18B
PLK1	3	19	
PMF1	3		
RCC2	2		TD60
SGOL1	1	2	
SGOL2	1	5	
SKA1	2	4	C18orf24
SKA	2	2	FAM33A
SKA3	2	9	C13orf3
SPC24	2	2	
SPC25	3	15	
STRA13	3		CENPX
TTK	3	15	
ZW10	2	6	
ZWILCH	2	6	
ZWINT	3	34	
Sum	155	422	
Average		6.59	

The genes that appeared more than 9 times in the combined co-expression list were summarized in Table 2. ZWINT, a well-characterized kinetochore protein, was represented 34 times in the combined list and ranked at the top [36]. Surprisingly, the second-ranking gene *TRIP13* has never previously been associated with mitosis regulation but will be further characterized in this work as a novel kinetochore component (see below). GO term enrichment analyses of the genes in Table 2 indicated significantly higher representation of genes functioning in mitosis (Additional file 4: Table S4). A few categories of genes participating in DNA replication were also enriched in the results. Despite the possibility that the co-expression search may have recovered genes generally important for cell proliferation, it should be noted that at least some of the genes (e.g. RFC complex subunits-encoding *CHTF18RFC3RFC4RFC5*) are critical for cohesion establishment during S phase [22,37]. As a further validation of the search strategy, we noticed certain genes in Table 2 have only been experimentally confirmed to participate in mitosis regulation in recent years, including those encoding centrosomal proteins STIL [38] and HMMR (RHAMM, [39]); centromere proteins RACGAP1 ([40]) and SUPT16H (Spt16 subunit of FACT complex, [41]); and other recently identified mitotic proteins NUSAP (involved in spindle organization, [42]) and CKAP2 (spindle function, [43]). This group can also be extended to include *ITGB3BP* (encoding CENP-R), *MLF1IP* (encoding CENP-U), *OIP5* (Mis18β), and *HJURP*, which all encode newly identified centromere proteins [44,45]. Although the later group of genes were among the 64 queries, they were so highly represented that they would still be retrieved if most of the constitutive centromere proteins (CENP-I to CENP-X) were omitted from queries. In addition, Table 2 and Additional file 3: Table S3 also contain several subunits of origin recognition complexes (*ORC1LORC2LORC4LORC5LORC6L*), γ-tubulin ring complexes (*TUBGCP2TUBGCP4TUBGCP5*), and condensin and cohesin complexes, supporting the roles of these complexes at kinetochores or in mitosis [24,46,47].

**Table 2:** Top-ranking genes that co-express with 64 core centromere-kinetochore components

**Genes**	**Occurrences in combined Gene Sorter "top 50" co-expression list**
ZWINT	34
TRIP13	31
BUB1B	30
CCNB2	30
CHEK1	27
KIF14	26
STIL	26
PTTG1	25
RRM1	25
ESPL1	24
NCAPH	24
ITGB3BP	23
KIF2C	23
RACGAP1	22
RFC4	22
FOXM1	21
BUB1	20
CCNB1	20
CKS1B	20
DBF4	20
FANCI	20
MLF1IP	20
NUSAP1	20
OIP5	20
TPX2	20
CDC2	19
CDCA8	19
CENPE	19
HMMR	19
MELK	19
PLK1	19
SMC4	19
TMPO	18
UBE2C	18
CDC45L	17
CDCA3	17
RFC3	17
ASF1B	16
CDKN3	16
KIF11	16
MCM2	16
PPIL5	16
RFC5	16
SPAG5	16
CDC25A	15
CDC6	15
CDCA5	15
PCNA	15
SPC25	15
TTK	15
CDK2	14
CENPA	14
GMPS	14
KIFC1	14
KPNA2	14
PBK	14
SMC2	14
TIMELESS	14
TYMS	14
CKAP2	13
DDX39	13
DTL	13
GMNN	13
MRPL39	13
NEK2	13
PSMG1	13
TTF2	13
WHSC1	13
ABCF1	12
IGF2BP3	12
MCM4	12
NDC80	12
POLE2	12
RNASEH2A	12
SUPT16H	12
UBE2T	12
AK129567	11
CENPF	11
CTPS	11
DLGAP5	11
DUT	11
KNTC1	11
MAD2L1	11
NUP155	11
PAK6	11
CCNA2	10
CDCA7	10
KIF20B	10
MCM3	10
WDR12	10
ARHGAP11A	9
AURKA	9
BLM	9
C13orf3	9
CHTF18	9
EZH2	9
GINS1	9
HJURP	9
MICB	9
NCAPD2	9
NUP107	9
NUP205	9
PAXIP1	9
PKMYT1	9
PPAT	9
TOP2A	9
TROAP	9
TSR1	9
TUBGCP4	9
Sum	1674
Average	15.36

The genes co-expressing with 64 centromere/kinetochore components were further analysed using the CoExSearch program which accepts a group of queries to search and rank common co-expressing genes [48,49] (see Methods for details). As seen in Additional file 5: Table S5, 37 out of the 64 genes are found among the top 300 genes (no gene chip data for *CENP-P* in CoExSearch). In addition, the CoExSearch and Gene Sorter “top 300” lists share 123 genes, with *TRIP13* among them (Additional file 6: Table S6). Due to coordination of many events in orchestrating mitosis progression, it should not be surprising that many among the 123 genes encode proteins that participate in different aspects of mitosis regulation, even though the query is a group of centromere/kinetochore proteins. Again, some among the 123 genes have only recently been associated with mitotic functions or structures such as *FANCD2FANCI* and *HYLS1*[50,51]. Future efforts will be directed to analyze those that still do not have defined mitotic functions (marked by “?” in Additional file 6: Table S6).

### Proteins interacting with 64 core centromere/kinetochore components

We then used POINeT website to obtain the “sub-network specific” PPI data with the 64 core centromere/kinetochore proteins collectively as a query group (see Methods for details) [52,53]. The tool was chosen mainly because it distinguishes a protein’s total interactors from the interactors within a “sub-network” (in this case determined by the group of 64 query genes). The tool partially solved the problem of retrieving too many “false positive” interactors that may share no functions with the queries. Fifty-eight out of 64 queries returned interactors, with 452 non-redundant PPIs involving 352 interactors (including queries) retrieved. Top ranked non-query proteins that tend to interact with the core centromere/kinetochore proteins is presented in Table 3 and a full list in Additional file 7: Table S7. The functional relevance of the ranking was supported by several lines of evidence. First, the first 21 and a total of 44 in the list encode query proteins, indicating the clustering of search results. Second, some non-query proteins such as different isoforms of regulatory B subunit of phosphatase 2A (two encoded by *PPP2R5A* and *PPP2R5D* in the list) were recently shown to localize at kinetochores and affect kinetochore-microtubule interactions [54,55]. In addition, many among the top-ranked non-query genes encode known centromere/kinetochore or mitotic regulatory proteins such as *PARP2CBX5* (encoding HP1α), *CCNB1* (encoding cyclin B1); microtubule subunits and associated proteins *TUBA4A* and *MAPRE2* (encoding EB2); proteins involved in mitotic ubiquitylation and regulation: *CDC16ANAPC7CDC27* (three APC/C subunits), *FBXO5* (encoding Emi1, an APC/C inhibitor), *MAD2L2* (another APC/C inhibitor) and *PSMA3* (a proteasome subunit).

**Table 3:** Top-ranking non-query genes whose encoded proteins interact with 64 core centromere-kinetochore components

**Ranking**	**Gene ID**	**Gene Official Symbol**	**Total interactions**	**Interactions with 64 queries**
22	10038	*PARP2*	6	3
36	10717	*AP4B1*	4	2
37	8881	*CDC16*	12	3
43	996	*CDC27*	26	4
47	51434	*ANAPC7*	9	2
48	26271	*FBXO5*	6	2
49	8546	*AP3B1*	12	2
50	5528	*PPP2R5D*	11	2
51	23468	*CBX5*	35	4
53	5494	*PPM1A*	16	2
54	9400	*RECQL5*	16	2
56	5525	*PPP2R5A*	21	2
57	10982	*MAPRE2*	8	2
58	675	*BRCA2*	28	2
59	891	*CCNB1*	35	2
60	51421	*AMOTL2*	11	2
61	57562	*KIAA1377*	81	4
62	163	*AP2B1*	33	2
63	22981	*NINL*	37	2
64	11335	*CBX3*	18	2
65	7277	*TUBA4A*	61	3
66	10459	*MAD2L2*	13	2
67	5684	*PSMA3*	37	2
68	890	*CCNA2*	36	2
69	324	*APC*	43	3
70	162	*AP1B1*	21	2
71	22919	*MAPRE1*	16	2
72	1874	*E2F4*	78	3
73	26258	*PLDN*	21	2
74	3066	*HDAC2*	85	3
75	142	*PARP1*	56	3
76	983	*CDC2*	131	4
77	5515	*PPP2CA*	59	2
78	7343	*UBTF*	27	2
79	81565	*NDEL1*	26	2
80	55290	*BRF2*	57	2
81	3065	*HDAC1*	162	5
82	5499	*PPP1CA*	82	3
83	1639	*DCTN1*	47	2
84	203068	*TUBB*	95	2
85	23043	*TNIK*	90	2
86	28964	*GIT1*	53	2
87	1869	*E2F1*	89	3
88	3692	*EIF6*	101	2
89	5921	*RASA1*	69	2
90	55183	*RIF1*	106	2
91	5764	*PTN*	86	2
92	8848	*TSC22D1*	103	2
93	11156	*PTP4A3*	101	2
94	7046	*TGFBR1*	160	2
95	5925	*RB1*	156	2
96	7428	*VHL*	208	2
97	7157	*TP53*	315	2
98	7189	*TRAF6*	369	2
99	7532	*YWHAG*	309	2

The mitotic functions of several other proteins are less well characterized but they were all reported in literature to interact with at least one of the 64 centromere/kinetochore proteins. Of interest, adapter proteins AP4B1, AP3B1 and AP2B1 interact with mitotic checkpoint kinases BUBR1 and BUB1, but the mitotic functions of the association have not been addressed [56]. However, clathrin has recently been shown to affect the spindle integrity during mitosis (for example, [57,58]), raising the possibility that these vesicle-trafficking proteins may indeed have mitotic functions. In addition, RECQL5 and BRCA2, two proteins involved in DNA repair, are of interest. BRCA2 has been localized at centrosomes and may affect genomic stability by altering centrosome behaviour [59]. Furthermore, KIAA1377 not only is localized at the midbody [52], but also interacts with kinetochore proteins ATRX, BMI1, CCDC99 (Spindly), MAD2L1BP (p31^comet^), PMF1 and other known mitosis regulators (http://www.ncbi.nlm.nih.gov/gene?term=kiaa1377). Interestingly, TRIP13, one of the top-ranked co-expressing genes, was also found to interact with p31^comet^ and several other mitosis regulators, although it did not make to the PPI top-ranking list mainly due to its large number of total interactors.

### Comparison of co-expression and PPI search results

Nineteen genes/proteins are ranked high in both Gene Sorter co-expression and POINeT PPI lists (Tables 2 and Additional file 7: Table S7), of which 16 were in the query list (*BUB1, BUB1B, CDCA8, CENP-A, CENP-E, CENP-F, ITGB3BP, KIF2C, KNTC1, MAD2L1, NDC80, NEK2, PLK1, SPC25, TTK* and *ZWINT*). The 3 non-query genes *CCNA2*, *CCNB1* and *CDC2* encode cyclin A2, cyclin B1 and CDC2 kinase. Similarly, comparison of co-expressing genes in Table S6 and PPI list in Table S7 found 21 common genes, with 18 in the query list (*BUB1, BUB1B, CDCA8, CENP-A, CENP-E, CENP-F, KIF2C, KNTC1, MAD2L1, NDC80, NEK2, NUF2, PLK1, SGOL2, SPC25, TTK, ZWILCH* and *ZWINT*) and 3 non-query genes: *CCNA2, CCNB1* and *BRCA2*. The convergence of results to well-known mitosis regulators is encouraging, reflecting that the searches have generated functionally relevant results. How much overlap one should expect from parallel co-expression and PPI analyses is hard to predict as the two analyses are based on information at mRNA and protein levels, respectively; and the PPI coverage of the proteome is usually much lower compared to transcriptional profiling for the genome.

It is acknowledged that high-throughput data contains intrinsic noises that affect both co-expression and PPI analyses [60-62]. In addition, many genes/proteins may participate in multiple biological processes. Using a group of functionally associated or subcellularly co-localized genes/proteins as queries has the potential to reduce database noises and selectively screen for candidates that are functional in a specific biological process or structure. Although we are mostly interested in using such “group query” strategy in combination with online resources to identify candidate mitosis regulators for experimental validation, refining the search strategy in collaboration with computer scientists and statisticians may prove useful to apply “partial knowledge” to obtain a more comprehensive understanding of a biological process or structure.

### TRIP13 is a novel kinetochore protein

To further validate the results from bioinformatics studies, subcellular localization was determined for seven candidate genes as preliminary evaluation of their potential mitotic functions. The cDNAs were cloned as GFP-fusions and were all confirmed to express at expected sizes (Additional file 8: Figure. S1).

As mentioned above, *TRIP13* is a top-ranking gene that co-expresses with centromere/kinetochore components, but has not been associated with any mitotic functions. The yeast, worm and mouse homologs of TRIP13 have all been implicated in meiosis recombination [63-65]. However, TRIP13 is widely expressed in somatic tissues (information from Gene Sorter). Moreover, proteomic studies have found TRIP13 interacts with p31^comet^, an important spindle assembly checkpoint silencing protein [66]. The interaction is conserved in both human and mouse cells [67,68]. TRIP13 encodes an AAA-ATPase, is overexpressed in many cancers and is hence listed in multiple cancer signatures [31,69-71].

**Figure 1:**
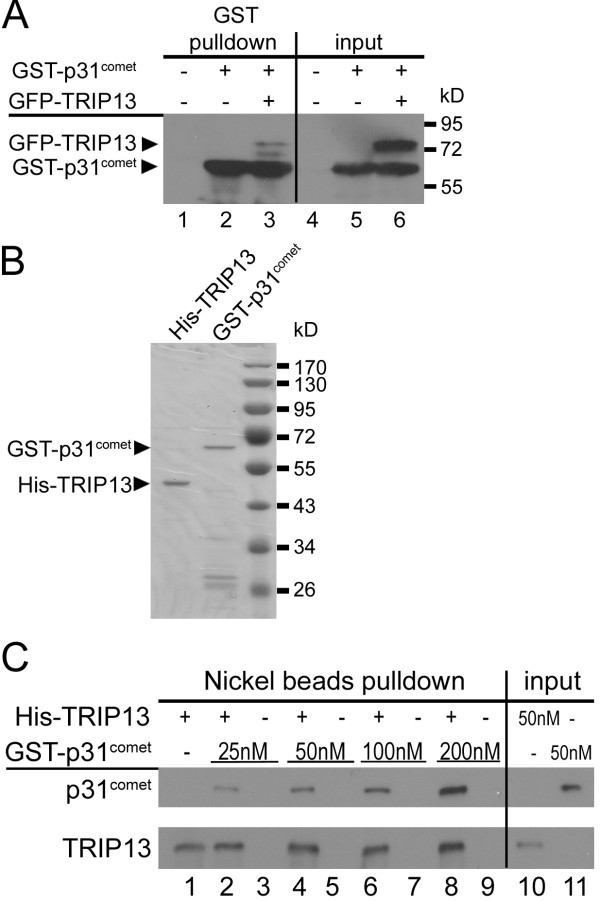
**Interaction between TRIP13 and p31**^**comet**^**.** (**A**) GST-p31^comet^ was expressed either alone or together with GFP-TRIP13 in asynchronous HEK293 cells. The input lysates and GST-pulldowns were probed with anti-GST and GFP antibodies. Untransfected lysates were used as controls. (**B**) Coomassie blue staining of SDS-PAGE gel loaded with ~0.4 μg of purified recombinant GST-p31^comet^ and His-tagged TRIP13. (**C**) *In vitro* binding assays using recombinant His-TRIP13 at 100 nM and GST-p31^comet^ at increasing concentrations. Proteins associated with washed nickel beads were probed with anti-6 × His and anti-GST antibodies.

**Figure 2:**
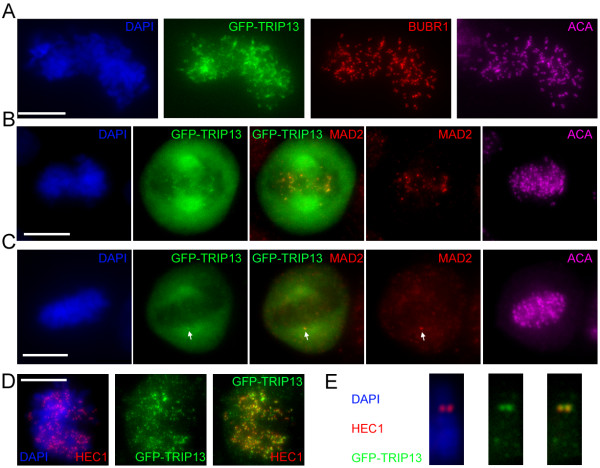
**Characterization of novel kinetochore protein TRIP13.** (**A**) A GFP-TRIP13 transfected HeLa cell in early prometaphase was stained for centromeres (ACA, purple), BUBR1 (red) and DNA (DAPI, blue) to compare with the GFP signals (green). Images shown are maximum projection of a z-series at 1 μm interval. Bar = 10 μm.(B&C) A GFP-TRIP13 transfected prometaphase cell (**B**) and a cell progressing into metaphase (**C**) were co-stained with anti-MAD2 antibody and ACA to show GFP-TRIP13 localization only at MAD2 positive kinetochores, such as the one indicated by arrows in (**C**).(**D**) A GFP-TRIP13 transfected cell spun onto a coverslip for mitotic chromosome spread preparation was probed with DAPI and anti-HEC1 antibody. (**E**) A single mitotic chromosome stained with DAPI, anti-GFP and anti-HEC1 antibodies to show kinetochore localization of GFP-TRIP13.

We first confirmed by GST-pulldown that GFP-TRIP13 associates with GST-p31^comet^ in cell lysates (Figure 1A). Furthermore, purified recombinant GST-p31^comet^ and His-TRIP13 directly interact *in vitro* under physiologically relevant concentrations (our estimates of endogenous concentrations of p31^comet^ and TRIP13 are both around 100 nM) (Figure 1B &C). In interphase cells GFP-TRIP13 is distributed in endoplasmic reticulum-like structures and partially localize at the nuclear envelope (data not shown). GFP-TRIP13 was observed to concentrate at kinetochores shortly after nuclear envelope breakdown, as evidenced by co-localization with BUBR1 and the centromere marker ACA (Figure 2A). In later prometaphase cells, GFP-TRIP13 was only observed at kinetochores that were also stained positive for SAC protein MAD2 (Figure 2B&C). Also similarly as MAD2, GFP-TRIP13 disappears from kinetochores in metaphase and anaphase cells (data not shown). The kinetochore localization of GFP-TRIP13 seems independent of microtubules, as it remains co-localized with HEC1/hNdc80 in chromosome spread preparations made from nocodazole and hypotonic buffer treated cells (Figure 2D&E). We concluded that TRIP13 is a novel kinetochore protein that interacts with p31^comet^. As SAC silencing requires energy input that utilizes hydrolysis of the β-γ phosphoanhydride bond in ATP [72,73], we are testing whether TRIP13 as an AAA-ATPase facilitates p31^comet^ mediated checkpoint silencing.

### KIAA1377 is a novel centrosomal protein

**Figure 3:**
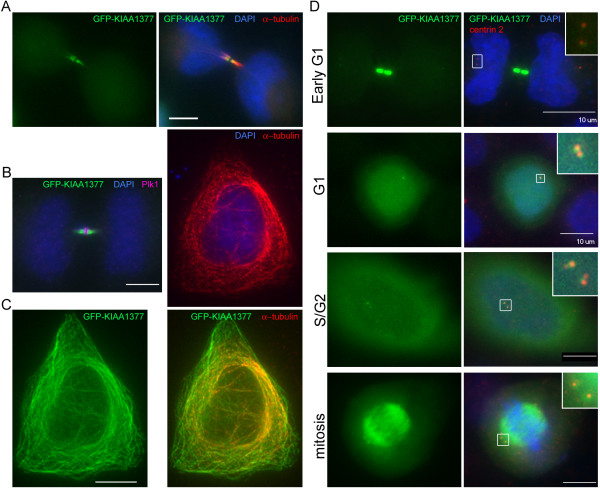
**Subcellular localization of GFP-KIAA1377.** HeLa cells transfected with GFP-KIAA1377 (green) were fixed and probed with immunofluorescence. Images at single focal planes are shown. Bar = 10 μm. (**A**&**B**) Transfected cells finishing cytokinesis were co-stained with anti-α-tubulin (red in A) or anti-Plk1 antibody (purple in B) and counter-stained with DAPI for DNA (blue). (**C**) A transfected cell expressing higher level of GFP-KIAA1377 (green) was co-stained for α-tubulin (red) and DNA (blue) to indicate co-localization of GFP-KIAA1377 with microtubule structures. (**D**) GFP-KIAA1377 is localized at centrosomes. Cells at different stages of cell cycle are distinguished based on anti-centrin 2 (red) and DAPI (blue) staining. The GFP signals in different cells were adjusted to the same scale. Enlarged insets show details of boxed areas

KIAA1377 was retrieved as a top-ranking non-query protein that associates with centromere/kinetochore proteins in the PPI analyses. KIAA1377 was previously shown to localize at the midbody [52]. GFP-KIAA1377 was confirmed to localize at midbody during cytokinesis, co-localizing with microtubule bundles adjacent to two Plk1-containing discs (Figure 3A&B). In cells showing relatively higher expression, GFP-KIAA1377 was also observed to extensively overlap with microtubule network in interphase cells and the spindle in mitotic cells (Figure 3C&D). Most interestingly, GFP-KIAA1377 co-localizes with centrin 2 at centrosomes throughout the cell cycle except on newly assembled daughter centrioles where GFP signals are absent (Figure 3D, S/G2). In early G1 cells, the signals of GFP-KIAA1377 at centrosomes are dim when compared to those at the midbody; nonetheless they are reproducibly detectable (Figure 3D, Early G1; Additional file 9: Figure S2, Early G1). Co-staining with γ-tubulin antibody confirmed the centrosome localization pattern of GFP-KIAA1377 (Additional file 9: Figure S2). The results indicate that KIAA1377 is likely a novel centrosomal protein. As mentioned above, KIAA1377 was reported in proteomics studies to also interact with p31^comet^. Studies are ongoing to further clarify potential mitotic functions of KIAA1377.

### Experimental characterization of other potential mitosis regulators

Five other co-transcription hits were also cloned as GFP-fusions and examined for their subcellular localization. Three were retrieved from both Gene Sorter and CoExSearch searches (Additonal file 6: Table S6), including *PBK* and *MELK* encoding protein kinases and *CDKN3* encoding dual specificity phosphatase KAP that is closely related to CDC14 phosphatases [74]. *DDX39*, ranked as the 61^st^ in the Gene Sorter search (Table 2) and encoding a RNA helicase, and *C4orf46*, a gene of unknown function and ranked as 32^nd^ in the CoExSearch result (Additional file 5; Table S5), were also included for analyses.

PBK/TOPK was previously shown to regulate cytokinesis [75]. KAP, originally discovered as a CDK inhibitor, interacts with CDK2 and CDC2 [76,77]. However, the subcellular localization of GFP-PBK and GFP-CDKN3 cannot be distinguished from that of GFP alone (Additional file 10: Figure S3). GFP is diffusely distributed in interphase cells with slightly higher accumulation in the nuclei. GFP alone was also observed to be concentrated at the midbody and, to a lesser extent, mitotic spindle (Additional file 10: Figure S3, first row). Lack of specific subcellular localization apparently does not preclude certain proteins from playing active roles during mitosis. A C-terminal tagged C4orf46-GFP shows similar localization in mitotic cells as GFP, GFP-PBK and GFP-CDKN3, although in interphase cells it is primarily localized in ER like structure and nuclear envelope (Additional file 10: Figure S3, bottom row).

**Figure 4:**
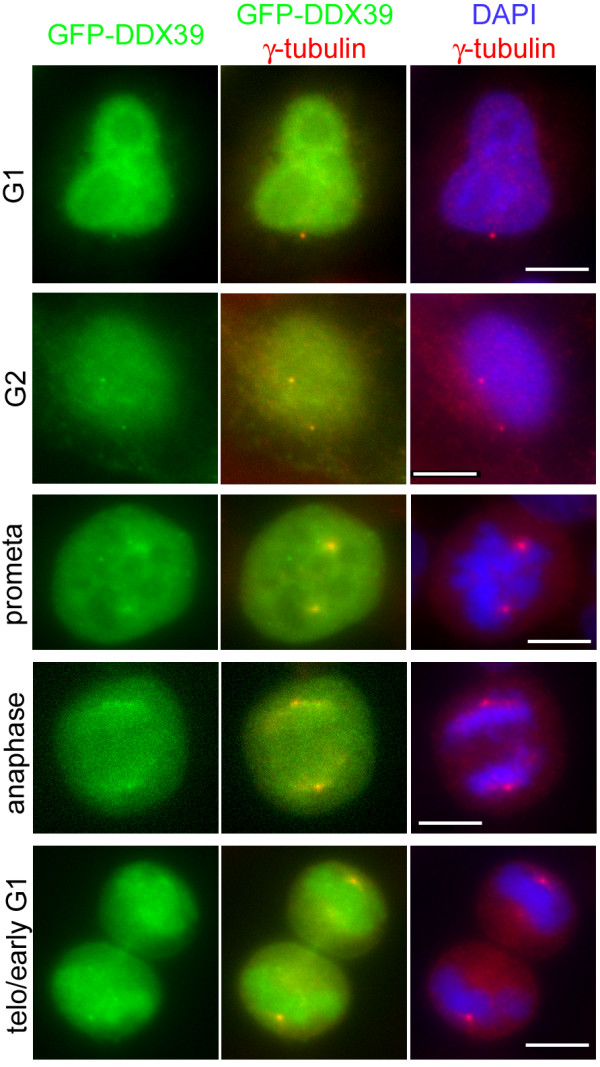
**GFP-DDX39 is a centrosomal protein.** Asynchronous HeLa cells were transfected with GFP-DDX39 (green), fixed and co-stained with DAPI (blue) and anti-γ-tubulin antibody (red). The cell cycle stages were assigned based on the numbers of γ-tubulin foci and DNA morphology. Bar = 10 μm.

**Figure 5:**
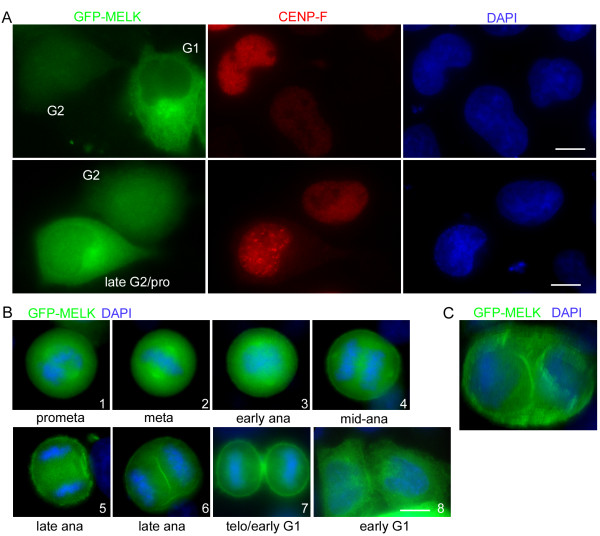
**Dynamic subcellular localization of GFP-MELK.** (**A**). Distribution of GFP-MELK signals in the cytoplasm and nuclei in G1 and G2 cells. G1 and G2 cells are differentiated based on characteristic staining patterns of CENP-F (red) [82]. Note nuclear signals of GFP-MELK in G2 cells are neither due to overexpression of GFP fusions (compare the G1 and G2 cells in the top row), nor affected by strong CENP-F staining in the Alexa Fluor 555 channel (compare nuclear GFP signals in G2 and late G2 cells in the bottom row). DNA was counterstained with DAPI. Bar = 10 μm. (**B**) Dynamic cortical localization of GFP-MELK throughout mitosis. Note the GFP signals at the midzone of a late anaphase cell (6). Bar = 10 μm. (**C**) A 3-D presentation of two GFP-MELK bands at the presumptive cleavage furrow surrounding two groups of separated chromosomes.

GFP-DDX39 and GFP-MELK displayed more interesting subcellular localization. Although the bulk of GFP-DDX39 is diffuse in both the cytoplasm and nuclei, a fraction of the GFP signals co-localizes with γ-tubulin throughout the cell cycle (Figure 4). Therefore, DDX39 is also a putative centrosomal protein and its possible functions at centrosomes or during mitosis warrant further investigation. It should be noted that RNA and RNA binding proteins have been found in centrosomes [78,79]. MELK was proposed to regulate G2/M transition by phosphorylating CDC25B [80]. More recently, *Xenopus* MELK was found to exhibit mitosis specific localization at the cell cortex and target to the presumptive site of cleavage furrow before any signs of ingression, suggesting a role in cytokinesis regulation [81]. Using nuclear localization of CENP-F as a G2 marker [82], we found that GFP-MELK is largely cytoplasmic in G1 cells, but partially translocated into nuclei in G2 cells (Figure 5A, top row). However, in late G2/early prophase cells where discrete CENP-F foci can be discerned, the nuclear level of GFP-MELK is reduced again (Figure 5A, bottom row). Similarly to what has been reported in the *Xenopus* system, GFP-MELK starts to accumulate at cell cortex upon mitosis entry and becomes more evident following metaphase-to-anaphase transition (Figure 5B, 1–4). Intriguingly, one or two transient GFP-MELK bands were observed in the midzone of late anaphase cells (Figure 5B, 5–6; Figure 5C), likely marking the presumptive cleavage site as in *Xenopus* cells. The bands coalesces as cytokinesis progresses but cortical association of GFP-MELK remains until sometime in early G1 (Figure 5B, 7–8). The dynamic localization pattern of GFP-MELK seems consistent with its proposed roles in G2/M transition and cytokinesis regulation. The apparent evolutionary conservation between *Xenopus* and human MELKs prompts further studies on their functioning mechanisms in regulating mitosis progression.

## Conclusions

In conclusion, we have compiled so far the most comprehensive list of centromere/kinetochore proteins in human cells. Data mining of gene expression and PPI databases using the centromere/kinetochore proteins as queries have retrieved candidate novel mitosis regulators. Experimental validation has discovered two novel centrosomal proteins KIAA1377 and DDX39, and one novel kinetochore protein TRIP13. Functional characterization of these proteins will likely reveal novel mechanisms of mitosis regulation. We conclude that transcriptional co-expression and PPI network analyses with known human centromere/kinetochore proteins as a query group help identify novel mitosis regulators.

## Methods

### Literature search and gene ontology analysis

The list of human centromere/kinetochore proteins were first derived from a previous review [16], and then updated through exhaustive abstract search in PubMed. Tracking the references during full-text literature review and screening through Gene Ontology (GO) website (http://www.geneontology.org/) also contributed to the compilation. The last amendment of the list was made on April 30, 2012. For GO analysis, the human genes annotated with “centromere” or “kinetochore” in the GO terms or IDs were filtered with "*H. Sapiens*" species filter and downloaded in the “gene association format” into Microsoft Excel. The conversion between gene symbols and IDs was carried out using Gene symbol Gene ID converter [83] (http://idconverter.bioinfo.cnio.es/). The GO enrichment analysis was performed using FuncAssociate 2.0 (http://llama.med.harvard.edu/funcassociate/).

### Transcriptional co-expression analysis

The transcriptional expression profiling data in the UCSC Human Gene Sorter (Mar. 2006 datasets) were used for transcriptional co-expression analysis [84]. The depository contains data of the human transcriptome in over 70 tissues and cell lines obtained on three microarray chips. Gene Sorter search returns each query with a list of genes ranked by similarity in expression patterns [85,86]. For each centromere/kinetochore query, the top 50 co-expressing genes on all chips were collected. A total of 155 “top 50” lists for the 64 core centromere/kinetochore components were then pooled. The Pivot Table function in Excel was used to count the occurrences of each gene in the combined list after removing queries.

The co-expression was also analyzed later using the CoExSearch program (http://coxpresdb.jp/top_search.shtml#CoExSearch) [48,49] which accepts a group of queries to search for common co-expressing genes and rank them based on a co-expression measure “mutual rank” (MR) [49]. A total of 4401 microarray expression datasets (no overlap with data in Gene Sorter) was used for human gene co-expression analysis.

### Protein-protein interaction network analysis

PPI network analysis was performed using online tools provided at the POINeT website (http://poinet.bioinformatics.tw) [52,53,87], using the 64 "core" centromere/kinetochore proteins as a query group. Only experimentally determined interactions were used to analyze the interactions. The POINeT website has imported data from several most popular PPI databases, and ranked the proteins based on a “subnetwork specificity score” (S3 score) reflecting their enrichment in a specific biological process defined by the query group. The scoring system consists of two parts. The first part examines the ratio of the sub-network degree to the global degree of any given node. In other words, it compares the number of PPIs (degree) between a protein (node) and members of the query group to the total number of PPIs involving the protein. The second part compares the number of PPIs involving a certain protein with members of the query group to the number of PPIs between the same protein and 1,000 randomly generated groups of the same size as the query group [52,53].

### Recombinant DNA, recombinant protein and In vitro protein binding assay

DNA cloning was performed using the Gateway system (Invitrogen) [88,89]. Full-length cDNAs encoding selected proteins were amplified and cloned into pENTR-TOPO vector. The constructs were verified for DNA sequences and then recombined into different destination vectors for protein expression in *E. coli* or mammalian cells. Recombinant GST-p31^comet^ and 6×His-TRIP13 were expressed and purified as described before [88,89]. *In vitro* binding assay with recombinant proteins was performed essentially as in [89] except that Probond nickel beads (Invitrogen) was used for pull-down.

### DNA transfection and immunofluorescence

DNA transfection and immunofluorescence was performed essentially according to [90]. HeLa-M or HEK293 cells were transfected using Fugene 6 (Roche) or TransIT –LT1 (Mirus) following the manufacturers’ instructions. Cells were usually fixed 24~48 hrs after transfection in 3.5 % paraformaldehyde for 7 min, extracted with KBT (10 mM Tris–HCl, pH7.5, 150 mM NaCl, 1 mg/ml BSA and 0.2 % Triton X-100) for 5 min, and then blocked with KB (KBT omitting Triton X-100) for at least 5 min prior to immunofluorescence. In cases that γ-tubulin was probed, the cells were fixed and extracted simultaneously in 3.5 % paraformaldehyde containing 1 % Triton X-100 for 7 min, and then blocked with KB. The list of antibodies used can be provided upon request. The images were collected by a cooled CCD camera (CoolSNAP HQ2; Photometrics) equipped on an automated Olympus IX-81 microscope using a PlanApo 60× NA 1.42 oil objective with z-step mostly set at 1 μm. Image acquisition and analysis were performed using Slidebook software (Intelligent Imaging Innovations) and further processed in Adobe Photoshop for presentations.

### Preparation of mitotic chromosome spreads

Transfected HeLa-M cells were harvested after 16 hr treatment with nocodazole (60 ng/ml final concentration) and swollen for 30 min in 75 mM KCl at room temperature. One millilitre of cell suspension was added to a 35 mm dish containing coverslips, and spun at 1,000 × g for 12 min in a Legend RT-Plus centrifuge (Thermo Scientific) on top of a 15 ml tube holder fitted inside a hanging bucket. The chromosome spreads were then fixed and processed for immunofluorescence.

## Abbreviations

PPI, Protein-protein interaction; SAC, Spindle assembly checkpoint; APC/C, Anaphase promoting complex/cyclosome; GO, Gene ontology; ACA, Anti-centromere autoimmune.

## Competing interests

The authors declared that they have no competing interests.

## Authors' contributions

STL conceived the study and performed imaging experiments. AT and STL performed literature search and analyzed retrieved results. AT, PO, SS, ZG and STL performed gene cloning and mammalian expression experiments. KW carried out recombinant protein expression and pulldown experiments. STL wrote the manuscript. All authors read and approved the final manuscript.

## Supplementary Material

Additional file 1 **Table S1.** Compilation of human centromere-kinetochore proteins. Click here for file

Additional file 2 **Table S2. **Comparison of the centromere/kinetochore list compiled in this work with GO annotated centromere/kinetochore genes. Click here for file

Additional file 3 **Table S3. **Summary table of all “top 50” genes that co-express with the 64 core centromere/kinetochore queries in Gene Sorter search. Click here for file

Additional file 4 **Table S4. **GO enrichment analysis of genes listed in Table.2**.** Click here for file

Additional file 5 **Table S5. **Top-ranking co-expressing genes retrieved by CoExSearch program. Click here for file

Additional file 6 **: Table S6. **Genes co-expressing with the 64 core centromere/kinetochore components in both Gene Sorter and CoExSearch searches. Highlighed genes were experimentally tested in this work. Click here for file

Additional file 7 **Table S7. **Top-ranking genes whose encoded proteins interact with the core centromere/kinetochore components. Click here for file

Additional file 8 **Figure S1. **Western blot of GFP-fusion proteins experimentally tested in this work. Except that C4orf46 was fused with a C-terminal GFP tag, all other constructs contain N-terminal GFP. Asynchronous HEK293 (for TRIP13 and KIAA) and HeLa cells (for the rest) were transfected and cell lysates were harvested 24 ~ 48 hrs later for anti-GFP Western blot. Click here for file

Additional file 9 **Figure S2. **Co-localization of GFP-KIAA1377 with γ-tubulin throughout the cell cycle. HeLa cells transfected with GFP-KIAA1377 were fixed and stained with DAPI (blue) and anti-γ-tubulin antibody (red). In the bottom row, note no bleedthrough of strong γ-tubulin signals to the green channel in the untransfected cell on the left. Bar = 10 μm. Click here for file

Additional file 10 **Figure S3. **Comparison of subcellular localization of GFP, GFP-CDKN3, GFP-PBK and C4orf46-GFP. Cells undergoing cytokinesis or in interphase or mitosis were probed. DNA is counterstained with DAPI (blue) and microtubules are stained with anti-α-tubulin antibody (red). Note microtubule staining is not always easily discernible because single focal plane images were shown, and the contrast is optimized to show the microtubule bundles at the midbody in cells undergoing cytokinesis. Bar = 10 μm. Click here for file

## References

[B1] Pines J Rieder CL Re-staging mitosis: a contemporary view of mitotic progression Nature cell biology 2001 3 1 E3 E6 10.1038/35050676 11146636

[B2] Muller GA Engeland K The central role of CDE/CHR promoter elements in the regulation of cell cycle-dependent gene transcription FEBS J 277 4 877 893 20015071 10.1111/j.1742-4658.2009.07508.x

[B3] Laoukili J Kooistra MR Bras A Kauw J Kerkhoven RM Morrison A Clevers H Medema RH FoxM1 is required for execution of the mitotic programme and chromosome stability Nature cell biology 2005 7 2 126 136 10.1038/ncb1217 15654331

[B4] Furuno N den Elzen N Pines J Human cyclin A is required for mitosis until mid prophase The Journal of cell biology 1999 147 2 295 306 10525536 10.1083/jcb.147.2.295 PMC2174228

[B5] Musacchio A Salmon ED The spindle-assembly checkpoint in space and time Nat Rev Mol Cell Biol 2007 8 5 379 393 17426725 10.1038/nrm2163

[B6] Potapova TA Daum JR Pittman BD Hudson JR Jones TN Satinover DL Stukenberg PT Gorbsky GJ The reversibility of mitotic exit in vertebrate cells Nature 2006 440 7086 954 958 16612388 10.1038/nature04652 PMC1513549

[B7] Cheeseman IM Desai A Molecular architecture of the kinetochore-microtubule interface Nature reviews 2008 9 1 33 46 10.1038/nrm2310 18097444

[B8] Chan GK Liu ST Yen TJ Kinetochore structure and function Trends Cell Biol 2005 15 11 589 598 16214339 10.1016/j.tcb.2005.09.010

[B9] Santaguida S Musacchio A The life and miracles of kinetochores EMBO J 2009 28 17 2511 2531 19629042 10.1038/emboj.2009.173 PMC2722247

[B10] Perpelescu M Fukagawa T The ABCs of CENPs Chromosoma 2011 120 5 425 446 21751032 10.1007/s00412-011-0330-0

[B11] Amor DJ Kalitsis P Sumer H Choo KH Building the centromere: from foundation proteins to 3D organization Trends Cell Biol 2004 14 7 359 368 15246429 10.1016/j.tcb.2004.05.009

[B12] Ohta S Bukowski-Wills JC Sanchez-Pulido L Alves Fde L Wood L Chen ZA Platani M Fischer L Hudson DF Ponting CP The protein composition of mitotic chromosomes determined using multiclassifier combinatorial proteomics Cell 2010 142 5 810 821 20813266 10.1016/j.cell.2010.07.047 PMC2982257

[B13] Cusick ME Klitgord N Vidal M Hill DE Interactome: gateway into systems biology Hum Mol Genet 2005 14 Spec No. 2 R171 R181 16162640 10.1093/hmg/ddi335

[B14] Rhodes DR Chinnaiyan AM Integrative analysis of the cancer transcriptome Nat Genet 2005 37 Suppl S31 S37 15920528 10.1038/ng1570

[B15] Eisen MB Spellman PT Brown PO Botstein D Cluster analysis and display of genome-wide expression patterns Proc Natl Acad Sci U S A 1998 95 25 14863 14868 9843981 10.1073/pnas.95.25.14863 PMC24541

[B16] Jablonski SA Liu ST Yen TJ Targeting the kinetochore for mitosis-specific inhibitors Cancer Biol Ther 2003 2 3 236 241 12878855 10.4161/cbt.2.3.384

[B17] Meraldi P McAinsh AD Rheinbay E Sorger PK Phylogenetic and structural analysis of centromeric DNA and kinetochore proteins Genome Biol 2006 7 3 R23 16563186 10.1186/gb-2006-7-3-r23 PMC1557759

[B18] Gorbsky GJ Ricketts WA Differential expression of a phosphoepitope at the kinetochores of moving chromosomes The Journal of cell biology 1993 122 6 1311 1321 7690762 10.1083/jcb.122.6.1311 PMC2119849

[B19] Ahonen LJ Kallio MJ Daum JR Bolton M Manke IA Yaffe MB Stukenberg PT Gorbsky GJ Polo-like kinase 1 creates the tension-sensing 3 F3/2 phosphoepitope and modulates the association of spindle-checkpoint proteins at kinetochores Curr Biol 2005 15 12 1078 1089 15964272 10.1016/j.cub.2005.05.026

[B20] Wong OK Fang G Plx1 is the 3 F3/2 kinase responsible for targeting spindle checkpoint proteins to kinetochores The Journal of cell biology 2005 170 5 709 719 16129782 10.1083/jcb.200502163 PMC2171348

[B21] Wong OK Fang G Cdk1 phosphorylation of BubR1 controls spindle checkpoint arrest and Plk1-mediated formation of the 3 F3/2 epitope The Journal of cell biology 2007 179 4 611 617 17998400 10.1083/jcb.200708044 PMC2080899

[B22] Peters JM Tedeschi A Schmitz J The cohesin complex and its roles in chromosome biology Genes Dev 2008 22 22 3089 3114 19056890 10.1101/gad.1724308

[B23] Hirano T Condensins: organizing and segregating the genome Curr Biol 2005 15 7 R265 R275 15823530 10.1016/j.cub.2005.03.037

[B24] Mishra RK Chakraborty P Arnaoutov A Fontoura BM Dasso M The Nup107-160 complex and gamma-TuRC regulate microtubule polymerization at kinetochores Nature cell biology 12 2 164 169 10.1038/ncb2016 PMC2859955 20081840

[B25] Ashburner M Ball CA Blake JA Botstein D Butler H Cherry JM Davis AP Dolinski K Dwight SS Eppig JT Gene ontology: tool for the unification of biology. The Gene Ontology Consortium Nat Genet 2000 25 1 25 29 10802651 10.1038/75556 PMC3037419

[B26] Steensgaard P Garre M Muradore I Transidico P Nigg EA Kitagawa K Earnshaw WC Faretta M Musacchio A Sgt1 is required for human kinetochore assembly EMBO reports 2004 5 6 626 631 15133482 10.1038/sj.embor.7400154 PMC1299074

[B27] Mukhopadhyay D Arnaoutov A Dasso M The SUMO protease SENP6 is essential for inner kinetochore assembly The Journal of cell biology 2010 188 5 681 692 20212317 10.1083/jcb.200909008 PMC2835930

[B28] Zhang B Horvath S A general framework for weighted gene co-expression network analysis Stat Appl Genet Mol Biol 2005 4 Article17 16646834 10.2202/1544-6115.1128

[B29] Benson M Breitling R Network theory to understand microarray studies of complex diseases Curr Mol Med 2006 6 6 695 701 17022739 10.2174/156652406778195044

[B30] Lee HK Hsu AK Sajdak J Qin J Pavlidis P Coexpression analysis of human genes across many microarray data sets Genome Res 2004 14 6 1085 1094 15173114 10.1101/gr.1910904 PMC419787

[B31] Rhodes DR Yu J Shanker K Deshpande N Varambally R Ghosh D Barrette T Pandey A Chinnaiyan AM Large-scale meta-analysis of cancer microarray data identifies common transcriptional profiles of neoplastic transformation and progression Proc Natl Acad Sci U S A 2004 101 25 9309 9314 15184677 10.1073/pnas.0401994101 PMC438973

[B32] Stuart JM Segal E Koller D Kim SK A gene-coexpression network for global discovery of conserved genetic modules Science (New York, NY 2003 302 5643 249 255 10.1126/science.1087447 12934013

[B33] Zhang W Morris QD Chang R Shai O Bakowski MA Mitsakakis N Mohammad N Robinson MD Zirngibl R Somogyi E The functional landscape of mouse gene expression J Biol 2004 3 5 21 15588312 10.1186/jbiol16 PMC549719

[B34] Ruan J Dean AK Zhang W A general co-expression network-based approach to gene expression analysis: comparison and applications BMC Syst Biol 4 8 20122284 10.1186/1752-0509-4-8 PMC2829495

[B35] Mefford D Mefford JA Enumerating the gene sets in breast cancer, a "direct" alternative to hierarchical clustering BMC Genomics 11 (1 482 20731868 10.1186/1471-2164-11-482 PMC2996978

[B36] Starr DA Saffery R Li Z Simpson AE Choo KH Yen TJ Goldberg ML HZwint-1, a novel human kinetochore component that interacts with HZW10 Journal of cell science 2000 113 Pt 11 1939 1950 10806105 10.1242/jcs.113.11.1939

[B37] Uhlmann F A matter of choice: the establishment of sister chromatid cohesion EMBO reports 2009 10 10 1095 1102 19745840 10.1038/embor.2009.207 PMC2744122

[B38] Kumar A Girimaji SC Duvvari MR Blanton SH Mutations in STIL, encoding a pericentriolar and centrosomal protein, cause primary microcephaly Am J Hum Genet 2009 84 2 286 290 19215732 10.1016/j.ajhg.2009.01.017 PMC2668020

[B39] Maxwell CA Keats JJ Crainie M Sun X Yen T Shibuya E Hendzel M Chan G Pilarski LM RHAMM is a centrosomal protein that interacts with dynein and maintains spindle pole stability Molecular biology of the cell 2003 14 6 2262 2276 12808028 10.1091/mbc.E02-07-0377 PMC194876

[B40] Lagana A Dorn JF De Rop V Ladouceur AM Maddox AS Maddox PS A small GTPase molecular switch regulates epigenetic centromere maintenance by stabilizing newly incorporated CENP-A Nature cell biology 2010 12 12 1186 1193 10.1038/ncb2129 21102442

[B41] Okada M Okawa K Isobe T Fukagawa T CENP-H-containing complex facilitates centromere deposition of CENP-A in cooperation with FACT and CHD1 Molecular biology of the cell 2009 20 18 3986 3995 19625449 10.1091/mbc.E09-01-0065 PMC2743618

[B42] Raemaekers T Ribbeck K Beaudouin J Annaert W Van Camp M Stockmans I Smets N Bouillon R Ellenberg J Carmeliet G NuSAP, a novel microtubule-associated protein involved in mitotic spindle organization The Journal of cell biology 2003 162 6 1017 1029 12963707 10.1083/jcb.200302129 PMC2172854

[B43] Seki A Fang G CKAP2 is a spindle-associated protein degraded by APC/C-Cdh1 during mitotic exit J Biol Chem 2007 282 20 15103 15113 17376772 10.1074/jbc.M701688200

[B44] Foltz DR Jansen LE Bailey AO Yates JR Bassett EA Wood S Black BE Cleveland DW Centromere-specific assembly of CENP-a nucleosomes is mediated by HJURP Cell 2009 137 3 472 484 19410544 10.1016/j.cell.2009.02.039 PMC2747366

[B45] Foltz DR Jansen LE Black BE Bailey AO Yates JR Cleveland DW The human CENP-A centromeric nucleosome-associated complex Nature cell biology 2006 8 5 458 469 10.1038/ncb1397 16622419

[B46] Nayak T Edgerton-Morgan H Horio T Xiong Y De Souza CP Osmani SA Oakley BR Gamma-tubulin regulates the anaphase-promoting complex/cyclosome during interphase The Journal of cell biology 190 3 317 330 20679430 10.1083/jcb.201002105 PMC2922653

[B47] Prasanth SG Prasanth KV Stillman B Orc6 involved in DNA replication, chromosome segregation, and cytokinesis Science (New York, NY 2002 297 5583 1026 1031 10.1126/science.1072802 12169736

[B48] Obayashi T Kinoshita K Rank of correlation coefficient as a comparable measure for biological significance of gene coexpression DNA Res 2009 16 5 249 260 19767600 10.1093/dnares/dsp016 PMC2762411

[B49] Obayashi T Hayashi S Shibaoka M Saeki M Ohta H Kinoshita K COXPRESdb: a database of coexpressed gene networks in mammals Nucleic Acids Res 2008 36 Database issue D77 D82 17932064 10.1093/nar/gkm840 PMC2238883

[B50] Chan KL Palmai-Pallag T Ying S Hickson ID Replication stress induces sister-chromatid bridging at fragile site loci in mitosis Nature cell biology 2009 11 6 753 760 10.1038/ncb1882 19465922

[B51] Dammermann A Pemble H Mitchell BJ McLeod I Yates JR Kintner C Desai AB Oegema K The hydrolethalus syndrome protein HYLS-1 links core centriole structure to cilia formation Genes Dev 2009 23 17 2046 2059 19656802 10.1101/gad.1810409 PMC2751977

[B52] Chen TC Lee SA Hong TM Shih JY Lai JM Chiou HY Yang SC Chan CH Kao CY Yang PC From midbody protein-protein interaction network construction to novel regulators in cytokinesis Journal of proteome research 2009 8 11 4943 4953 19799413 10.1021/pr900325f

[B53] Chen TC Lee SA Chan CH Juang YL Hong YR Huang YH Lai JM Kao CY Huang CY Cliques in mitotic spindle network bring kinetochore-associated complexes to form dependence pathway Proteomics 2009 9 16 4048 4062 19658104 10.1002/pmic.200900231

[B54] Kitajima TS Sakuno T Ishiguro K Iemura S Natsume T Kawashima SA Watanabe Y Shugoshin collaborates with protein phosphatase 2A to protect cohesin Nature 2006 441 7089 46 52 16541025 10.1038/nature04663

[B55] Foley EA Maldonado M Kapoor TM Formation of stable attachments between kinetochores and microtubules depends on the B56-PP2A phosphatase Nature cell biology 2011 13 10 1265 1271 10.1038/ncb2327 PMC3186838 21874008

[B56] Cayrol C Cougoule C Wright M The beta2-adaptin clathrin adaptor interacts with the mitotic checkpoint kinase BubR1 Biochem Biophys Res Commun 2002 298 5 720 730 12419313 10.1016/s0006-291x(02)02522-6

[B57] Royle SJ Bright NA Lagnado L Clathrin is required for the function of the mitotic spindle Nature 2005 434 7037 1152 1157 15858577 10.1038/nature03502 PMC3492753

[B58] Lin CH Hu CK Shih HM Clathrin heavy chain mediates TACC3 targeting to mitotic spindles to ensure spindle stability The Journal of cell biology 2010 189 7 1097 1105 20566684 10.1083/jcb.200911120 PMC2894451

[B59] Nakanishi A Han X Saito H Taguchi K Ohta Y Imajoh-Ohmi S Miki Y Interference with BRCA2, which localizes to the centrosome during S and early M phase, leads to abnormal nuclear division Biochem Biophys Res Commun 2007 355 1 34 40 17286961 10.1016/j.bbrc.2007.01.100

[B60] Deane CM Salwinski L Xenarios I Eisenberg D Protein interactions: two methods for assessment of the reliability of high throughput observations Mol Cell Proteomics 2002 1 5 349 356 12118076 10.1074/mcp.m100037-mcp200

[B61] Bader JS Chaudhuri A Rothberg JM Chant J Gaining confidence in high-throughput protein interaction networks Nat Biotechnol 2004 22 1 78 85 14704708 10.1038/nbt924

[B62] Draghici S Khatri P Eklund AC Szallasi Z Reliability and reproducibility issues in DNA microarray measurements Trends Genet 2006 22 2 101 109 16380191 10.1016/j.tig.2005.12.005 PMC2386979

[B63] Li XC Schimenti JC Mouse pachytene checkpoint 2 (trip13) is required for completing meiotic recombination but not synapsis PLoS Genet 2007 3 8 e130 17696610 10.1371/journal.pgen.0030130 PMC1941754

[B64] Roig I Dowdle JA Toth A de Rooij DG Jasin M Keeney S Mouse TRIP13/PCH2 is required for recombination and normal higher-order chromosome structure during meiosis PLoS Genet 2010 6 8 10.1371/journal.pgen.1001062 PMC2920839 20711356

[B65] Wojtasz L Daniel K Roig I Bolcun-Filas E Xu H Boonsanay V Eckmann CR Cooke HJ Jasin M Keeney S Mouse HORMAD1 and HORMAD2, two conserved meiotic chromosomal proteins, are depleted from synapsed chromosome axes with the help of TRIP13 AAA-ATPase PLoS Genet 2009 5 10 e1000702 19851446 10.1371/journal.pgen.1000702 PMC2758600

[B66] Habu T Kim SH Weinstein J Matsumoto T Identification of a MAD2-binding protein, CMT2, and its role in mitosis EMBO J 2002 21 23 6419 6428 12456649 10.1093/emboj/cdf659 PMC136962

[B67] Rual JF Venkatesan K Hao T Hirozane-Kishikawa T Dricot A Li N Berriz GF Gibbons FD Dreze M Ayivi-Guedehoussou N Towards a proteome-scale map of the human protein-protein interaction network Nature 2005 437 7062 1173 1178 16189514 10.1038/nature04209

[B68] Stelzl U Worm U Lalowski M Haenig C Brembeck FH Goehler H Stroedicke M Zenkner M Schoenherr A Koeppen S A human protein-protein interaction network: a resource for annotating the proteome Cell 2005 122 6 957 968 16169070 10.1016/j.cell.2005.08.029

[B69] Yasugi T Vidal M Sakai H Howley PM Benson JD Two classes of human papillomavirus type 16 E1 mutants suggest pleiotropic conformational constraints affecting E1 multimerization, E2 interaction, and interaction with cellular proteins J Virol 1997 71 8 5942 5951 9223484 10.1128/jvi.71.8.5942-5951.1997 PMC191850

[B70] Carter SL Eklund AC Kohane IS Harris LN Szallasi Z A signature of chromosomal instability inferred from gene expression profiles predicts clinical outcome in multiple human cancers Nat Genet 2006 38 9 1043 1048 16921376 10.1038/ng1861

[B71] Martin KJ Patrick DR Bissell MJ Fournier MV Prognostic breast cancer signature identified from 3D culture model accurately predicts clinical outcome across independent datasets PLoS One 2008 3 8 e2994 18714348 10.1371/journal.pone.0002994 PMC2500166

[B72] Miniowitz-Shemtov S Teichner A Sitry-Shevah D Hershko A ATP is required for the release of the anaphase-promoting complex/cyclosome from inhibition by the mitotic checkpoint Proc Natl Acad Sci U S A 2010 107 12 5351 5356 20212161 10.1073/pnas.1001875107 PMC2851749

[B73] Teichner A Eytan E Sitry-Shevah D Miniowitz-Shemtov S Dumin E Gromis J Hershko A p31comet promotes disassembly of the mitotic checkpoint complex in an ATP-dependent process Proc Natl Acad Sci U S A 2011 108 8 3187 3192 21300909 10.1073/pnas.1100023108 PMC3044357

[B74] Patterson KI Brummer T O'Brien PM Daly RJ Dual-specificity phosphatases: critical regulators with diverse cellular targets Biochem J 2009 418 3 475 489 19228121 10.1042/bj20082234

[B75] Abe Y Takeuchi T Kagawa-Miki L Ueda N Shigemoto K Yasukawa M Kito K A mitotic kinase TOPK enhances Cdk1/cyclin B1-dependent phosphorylation of PRC1 and promotes cytokinesis J Mol Biol 2007 370 2 231 245 17512944 10.1016/j.jmb.2007.04.067

[B76] Hannon GJ Casso D Beach D KAP: a dual specificity phosphatase that interacts with cyclin-dependent kinases Proc Natl Acad Sci U S A 1994 91 5 1731 1735 8127873 10.1073/pnas.91.5.1731 PMC43237

[B77] Poon RY Hunter T Dephosphorylation of Cdk2 Thr160 by the cyclin-dependent kinase-interacting phosphatase KAP in the absence of cyclin Science (New York, NY 1995 270 5233 90 93 10.1126/science.270.5233.90 7569954

[B78] Song MH Aravind L Muller-Reichert T O'Connell KF The conserved protein SZY-20 opposes the Plk4-related kinase ZYG-1 to limit centrosome size Developmental cell 2008 15 6 901 912 19081077 10.1016/j.devcel.2008.09.018 PMC2829447

[B79] Alliegro MC Alliegro MA Centrosomal RNA correlates with intron-poor nuclear genes in Spisula oocytes Proc Natl Acad Sci U S A 2008 105 19 6993 6997 18458332 10.1073/pnas.0802293105 PMC2383940

[B80] Davezac N Baldin V Blot J Ducommun B Tassan JP Human pEg3 kinase associates with and phosphorylates CDC25B phosphatase: a potential role for pEg3 in cell cycle regulation Oncogene 2002 21 50 7630 7641 12400006 10.1038/sj.onc.1205870

[B81] Le Page Y Chartrain I Badouel C Tassan JP A functional analysis of MELK in cell division reveals a transition in the mode of cytokinesis during Xenopus development Journal of cell science 2011 124 Pt 6 958 968 21378312 10.1242/jcs.069567

[B82] Liao H Winkfein RJ Mack G Rattner JB Yen TJ CENP-F is a protein of the nuclear matrix that assembles onto kinetochores at late G2 and is rapidly degraded after mitosis J Cell Biol 1995 130 507 518 7542657 10.1083/jcb.130.3.507 PMC2120529

[B83] Alibes A Yankilevich P Canada A Diaz-Uriarte R IDconverter and IDClight: conversion and annotation of gene and protein IDs BMC Bioinforma 2007 8 9 10.1186/1471-2105-8-9 PMC1779800 17214880

[B84] Kent WJ Hsu F Karolchik D Kuhn RM Clawson H Trumbower H Haussler D Exploring relationships and mining data with the UCSC Gene Sorter Genome Res 2005 15 5 737 741 15867434 10.1101/gr.3694705 PMC1088302

[B85] Su AI Cooke MP Ching KA Hakak Y Walker JR Wiltshire T Orth AP Vega RG Sapinoso LM Moqrich A Large-scale analysis of the human and mouse transcriptomes Proc Natl Acad Sci U S A 2002 99 7 4465 4470 11904358 10.1073/pnas.012025199 PMC123671

[B86] Su AI Wiltshire T Batalov S Lapp H Ching KA Block D Zhang J Soden R Hayakawa M Kreiman G A gene atlas of the mouse and human protein-encoding transcriptomes Proc Natl Acad Sci U S A 2004 101 16 6062 6067 15075390 10.1073/pnas.0400782101 PMC395923

[B87] Lee SA Chan CH Chen TC Yang CY Huang KC Tsai CH Lai JM Wang FS Kao CY Huang CY POINeT: protein interactome with sub-network analysis and hub prioritization BMC Bioinforma 2009 10 114 10.1186/1471-2105-10-114 PMC2683814 19379523

[B88] Tipton AR Tipton M Yen T Liu ST Closed MAD2 (C-MAD2) is selectively incorporated into the mitotic checkpoint complex (MCC) Cell cycle (Georgetown, Tex 2011 10 21 3740 3750 10.4161/cc.10.21.17919 PMC3266009 22037211

[B89] Tipton AR Wang K Link L Bellizzi JJ Huang H Yen T Liu ST BUBR1 and Closed MAD2 (C-MAD2) Interact Directly to Assemble a Functional Mitotic Checkpoint Complex J Biol Chem 2011 286 24 21173 21179 21525009 10.1074/jbc.M111.238543 PMC3122179

[B90] Liu ST Hittle JC Jablonski SA Campbell MS Yoda K Yen TJ Human CENP-I specifies localization of CENP-F, MAD1 and MAD2 to kinetochores and is essential for mitosis Nature cell biology 2003 5 4 341 345 10.1038/ncb953 12640463

