# Supplementary figures and images for: Identification of novel mitosis regulators through data mining with human centromere/kinetochore proteins as group queries
Source: BMC Cell Biol. 2012 Jun 19;13:15. doi: 10.1186/1471-2121-13-15 (PMC3419070; doi:10.1186/1471-2121-13-15)

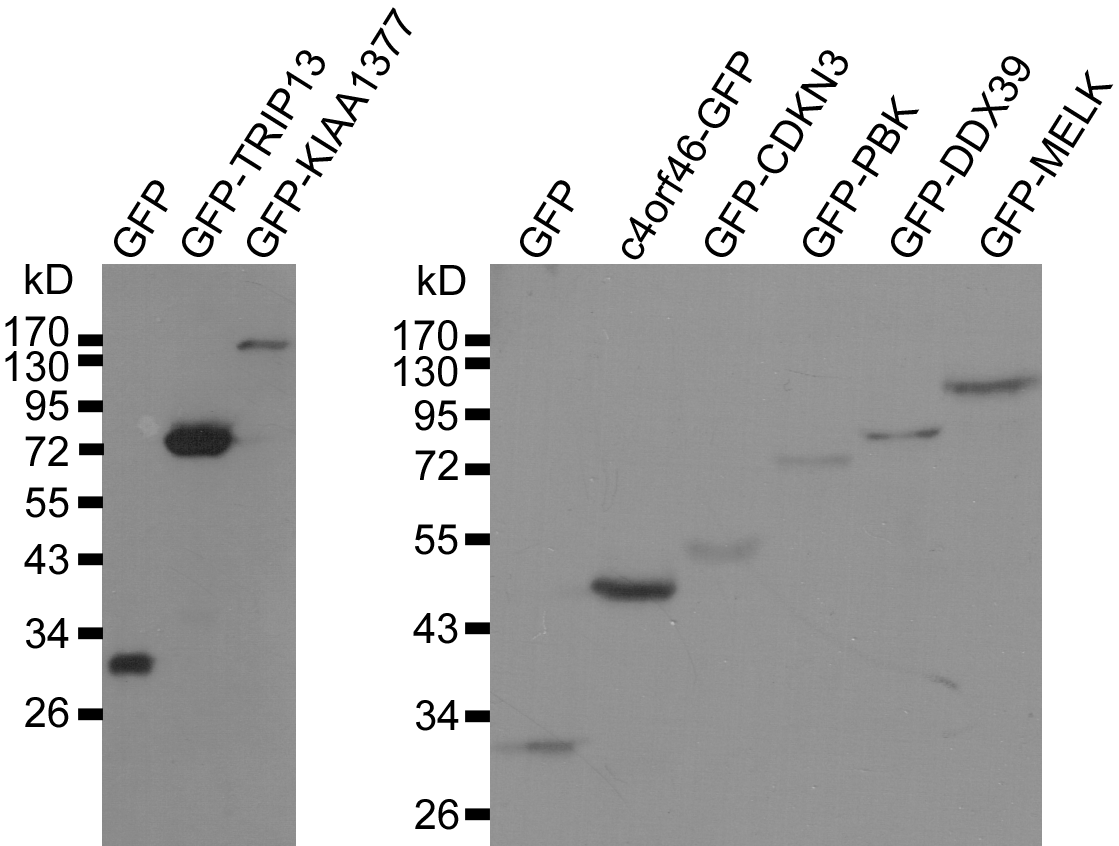

Supplement: Additional file 8 — Figure S1. Western blot of GFP-fusion proteins experimentally tested in this work. Except that C4orf46 was fused with a C-terminal GFP tag, all other constructs contain N-terminal GFP. Asynchronous HEK293 (for TRIP13 and KIAA) and HeLa cells (for the rest) were transfected and cell lysates were harvested 24 ~ 48 hrs later for anti-GFP Western blot. [file 1471-2121-13-15-S8.tiff]

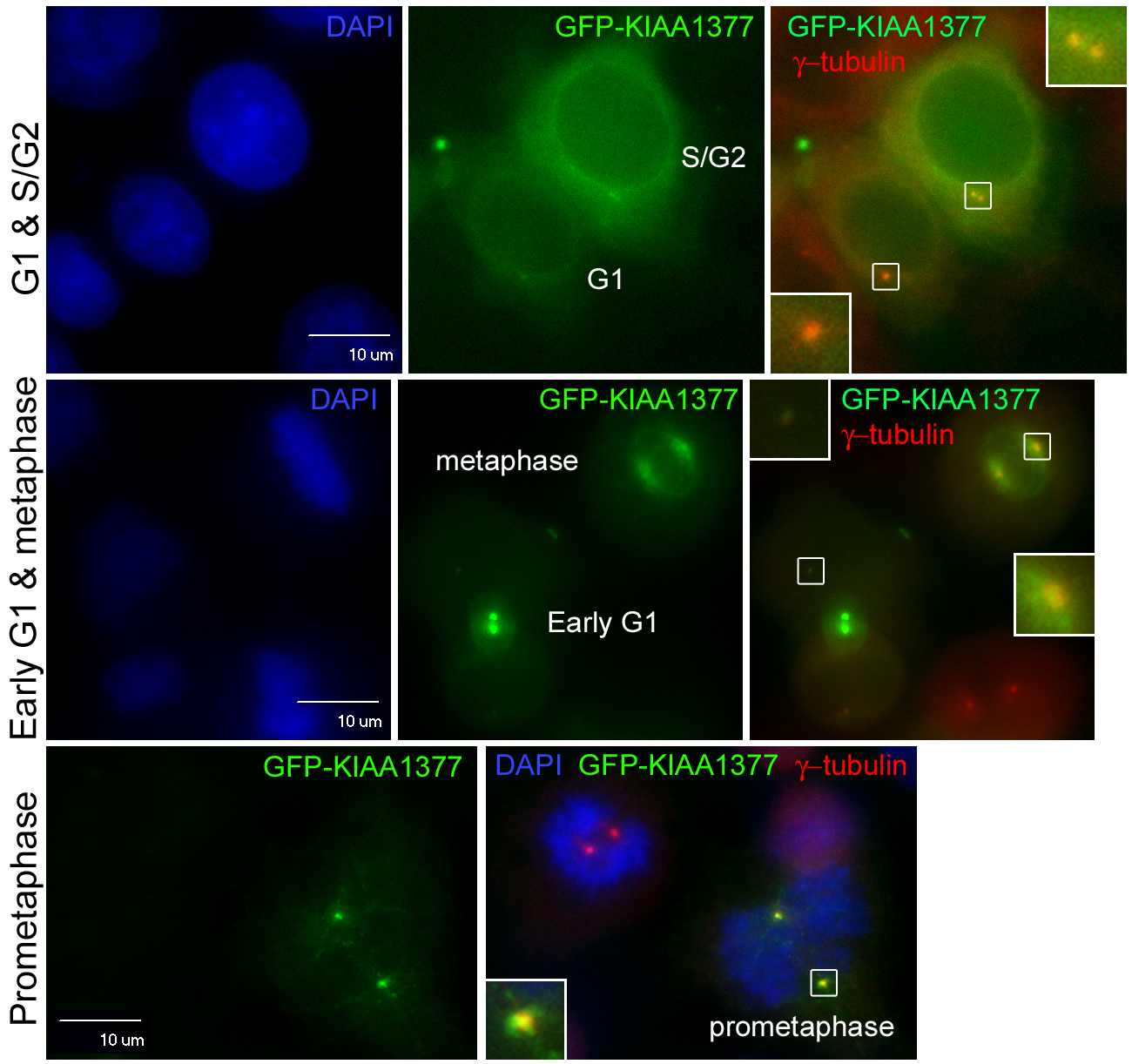

Supplement: Additional file 9 — Figure S2. Co-localization of GFP-KIAA1377 with γ-tubulin throughout the cell cycle. HeLa cells transfected with GFP-KIAA1377 were fixed and stained with DAPI (blue) and anti-γ-tubulin antibody (red). In the bottom row, note no bleedthrough of strong γ-tubulin signals to the green channel in the untransfected cell on the left. Bar = 10 μm. [file 1471-2121-13-15-S9.tiff]

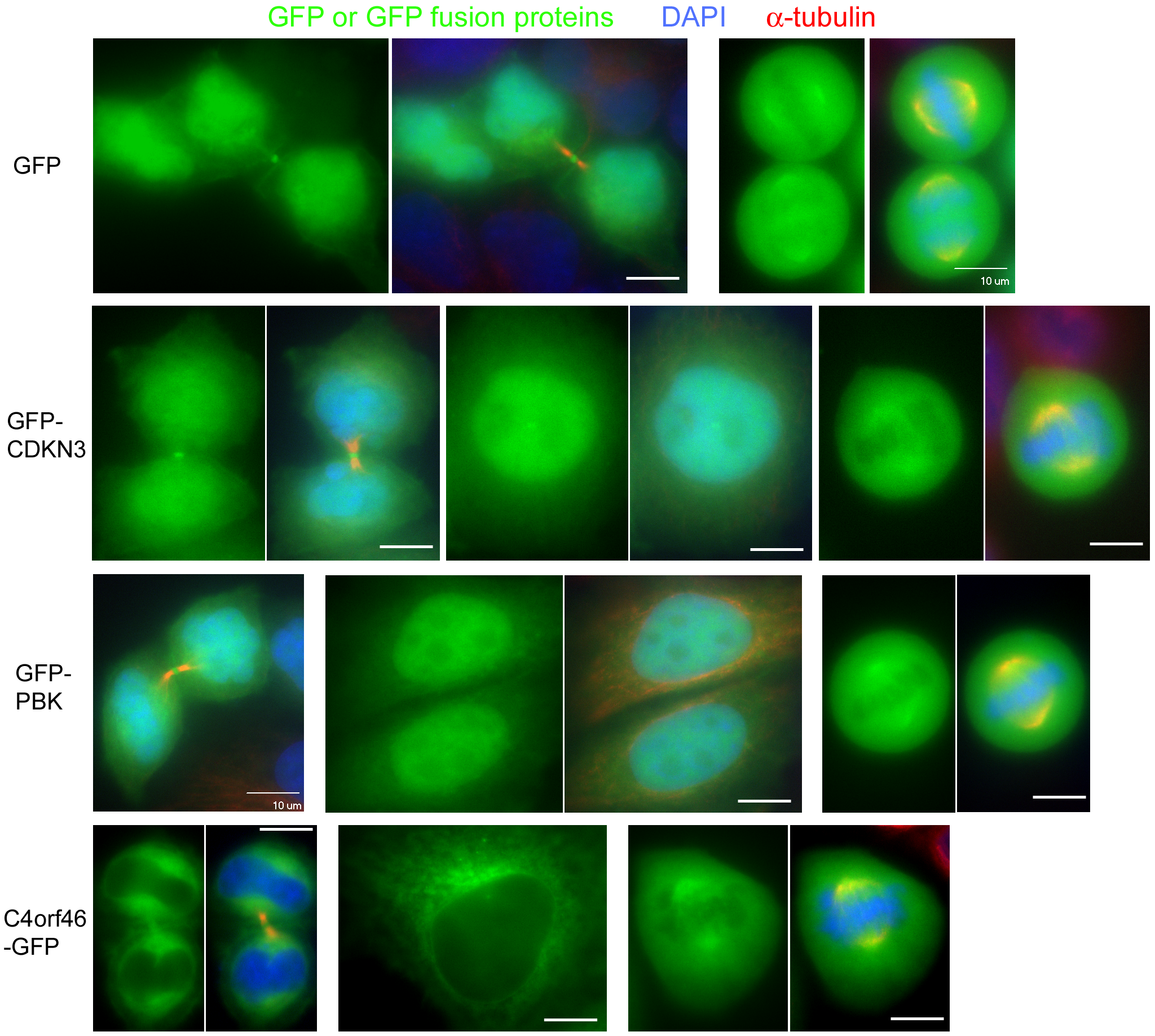

Supplement: Additional file 10 — Figure S3. Comparison of subcellular localization of GFP, GFP-CDKN3, GFP-PBK and C4orf46-GFP. Cells undergoing cytokinesis or in interphase or mitosis were probed. DNA is counterstained with DAPI (blue) and microtubules are stained with anti-α-tubulin antibody (red). Note microtubule staining is not always easily discernible because single focal plane images were shown, and the contrast is optimized to show the microtubule bundles at the midbody in cells undergoing cytokinesis. Bar = 10 μm. [file 1471-2121-13-15-S10.tiff]
